# Towards more efficient use of intravenous lumens in multi-infusion settings: development and evaluation of a multiplex infusion scheduling algorithm

**DOI:** 10.1186/s12911-020-01231-w

**Published:** 2020-09-02

**Authors:** Frank Doesburg, Roy Oelen, Maurits H. Renes, Wouter Bult, Daan J. Touw, Maarten W. Nijsten

**Affiliations:** 1 Department of Critical Care, University of Groningen, University Medical Center Groningen, Hanzeplein 1, Groningen, 9713 GZ The Netherlands; 2 Department of Clinical Pharmacy and Pharmacology, University of Groningen, University Medical Center Groningen, Groningen, The Netherlands; 3 Department of Pharmaceutical Analysis, University of Groningen, Groningen Research Institute of Pharmacy, Groningen, The Netherlands

**Keywords:** Infusions, intravenous, Algorithms, Infusion pumps, Injection site reaction, Drug incompatibility

## Abstract

**Background:**

Multi-drug intravenous (IV) therapy is one of the most common medical procedures used in intensive care units (ICUs), operating rooms, oncology wards and many other hospital departments worldwide. As drugs or their solvents are frequently chemically incompatible, many solutions must be administered through separate lumens. When the number of available lumens is too low to facilitate the safe administration of these solutions, additional (peripheral) IV catheters are often required, causing physical discomfort and increasing the risk for catheter related complications. Our objective was to develop and evaluate an algorithm designed to reduce the number of intravenous lumens required in multi-infusion settings by multiplexing the administration of various parenteral drugs and solutions.

**Methods:**

A multiplex algorithm was developed that schedules the alternating IV administration of multiple incompatible IV solutions through a single lumen, taking compatibility-related, pharmacokinetic and pharmacodynamic constraints of the relevant drugs into account. The conventional scheduling procedure executed by ICU nurses was used for comparison. The number of lumens required by the conventional procedure (L_CONV_) and multiplex algorithm (L_MX_) were compared.

**Results:**

We used data from 175,993 ICU drug combinations, with 2251 unique combinations received by 2715 consecutive ICU patients. The mean ± SD number of simultaneous IV solutions was 2.8 ± 1.6. In 27% of all drug combinations, and 61% of the unique combinations the multiplex algorithm required fewer lumens (*p* < 0.001). With increasing L_CONV_, the reduction in number of lumens by the multiplex algorithm further increased (*p* < 0.001). In only 1% of cases multiplexing required > 3 lm, versus 12% using the conventional procedure.

**Conclusion:**

The multiplex algorithm addresses a major issue that occurs in ICUs, operating rooms, oncology wards, and many other hospital departments where several incompatible drugs are infused through a restricted number of lumens. The multiplex algorithm allows for more efficient use of IV lumens compared to the conventional multi-infusion strategy.

## Background

Intravenous (IV) therapy is one of the most common treatment modalities in hospitals worldwide. Utilizing an infusion pump, solutions are typically delivered into the bloodstream at a preset and fixed rate. In the intensive care unit (ICU), operating rooms, and in oncology wards patients usually receive multiple IV solutions simultaneously from multiple infusion pumps. As drugs or their solvents are frequently chemically incompatible, many solutions must be administered through separate lumens in order to avoid precipitation or inactivation of components. When the number of available lumens is too low to facilitate the safe administration of these solutions, additional (peripheral) IV catheters are often required, causing physical discomfort, increasing the risk for catheter related complications, increased workload and associated treatment costs [1–4].

**Fig. 1:**
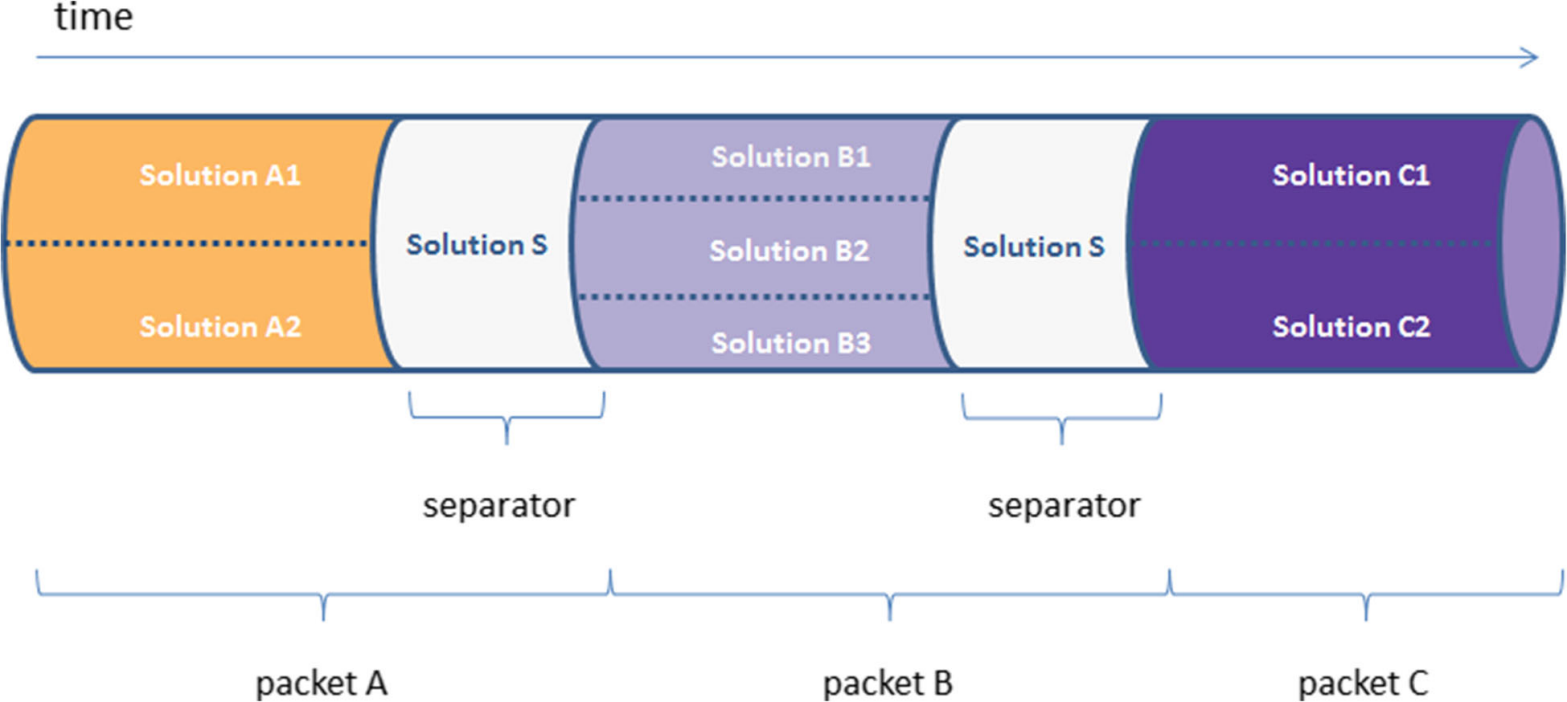
Multiplexed fluid administration through an IV tube. Using multiplex infusion packets of intravenous solutions **a**, **b**, and **c** are administered through a single IV tube, where solution S serves as separator. All drugs within a packet are compatible with each other

In order to circumvent these drug incompatibility issues we propose a novel administration method called multiplex infusion. Using this method, incompatible solutions are sequentially administered through the same lumen as infusion packets, while being separated by another solution that is compatible with both infusion packets (Fig. 1). In order to facilitate the timed alternation of these pumps, a centralized control system is required that generates an administration schedule and takes care of its execution by sending the appropriate commands to the infusion pumps at the bedside [5]. Multiplex infusion or multiplexing requires many switching actions between infusion pumps that cannot be reliably performed manually. An important time constraint for drug multiplexing is the maximally allowable interruption time (T_iMax_) between two administrations of the same drug. If the administration of a drug is interrupted too long, plasma or tissue concentrations may decrease to a point where the drug is no longer effective [6]. Therefore vasoactive drugs with a very short half-life (T_1/2_) such as norepinephrine with a T_1/2_ < 2.5 min [7], are considered not suitable for interrupted administration. Another important constraint is whether two drugs are compatible with each other, which determines whether or not multiple drugs can be administered simultaneously in a single infusion packet.

Scheduling algorithms are used in a broad spectrum of complex applications that rely on computer control, such as nuclear power plants, automotive systems and air traffic control [8]. In their seminal paper Scheduling Algorithms for Multiprogramming in a Hard-Real-Time Environment, Liu and Layland in 1973 described the earliest deadline first (EDF) scheduling algorithm for a set of periodically recurring tasks to be performed by a computer processor [9]. In the original EDF algorithm, every instance (i) of a task is associated with a duration of time required to complete the task (D_i_) and a period of time in which an instance of that task should be scheduled (P_i_). EDF scheduling is characterized by the prioritization of the tasks with the nearest deadline, i.e. nearest to the end of the P_i_. By analogy, multiplexed administrations of drugs can be regarded as a set of periodically recurring tasks that are processed by a single processor (an IV lumen), where every drug is a task that must be administered for a certain amount of time within a limited time frame. In the following sections we describe a multiplex scheduling algorithm that is designed to reduce the number of intravenous (IV) lumens required in multi-infusion settings that incorporates EDF scheduling [10]. We evaluated the performance of this algorithm by comparing the number of IV lumens required by conventional scheduling of therapeutic drugs with scheduling by the multiplex scheduling algorithm in a large real-life dataset.

## Methods

The goal of this study was to develop and evaluate the performance of a multiplex algorithm designed to reduce the number of IV lumens required in multi-infusion settings. To quantify the performance of a multiplex scheduling algorithm relative to conventional practice, we used the number of lumens required for the administration of therapeutic IV drugs as the outcome measure. For this purpose, we also modelled the conventional procedure that ICU nurses follow to combine IV drugs using one or more IV lumens for drugs to be administered both continuously and concurrently. The input for this model is a list of drugs to be administered and a database containing both drug characteristics and y-site drug compatibilities [9]. The output of this model is a distribution of drugs to be administered through a number of lumens.

The multiplex scheduling algorithm takes drug-specific time constraints into account for drugs that are multiplexed. Drugs that are not allowed to be multiplexed (e.g. norepinephrine) are scheduled using the conventional scheduling procedure. Thus, the output of the multiplex algorithm is a distribution of drugs to be multiplexed through a single lumen and a distribution of remaining drugs to be administered continuously through an additional number of lumens.

### Scheduling input

In a parallel research project, PK/PD drug properties of frequently used drugs in ICU were gathered from research literature, simulations using MWPharm v3.81 (MEDIWARE Inc., Groningen, Netherlands) software and subsequently expert assessment by pharmacists and intensivists (MHR, WB, DJT and MWN) (Table 1). These data include biological half-life, maximally allowable interruption time and whether multiplexed administration is allowed. Drug compatibility data were gathered from a local compatibility chart (Additional file 1) and a local parenteral drug guide, that in turn is derived from the IBM Micromedex database (IBM corporation, Armonk, United States of America), summary of product characteristics and the KNMP Kennisbank [11].

**Table 1:** Drug multiplexing parameters

Drug name	Multiplexing allowed	BT_**1/2**_ (min)	Maximal interruption time (min)^**a**^	Maximal administration rate (mg/min unless otherwise specified)^**b**^	ICU Concentration (mg/ml unless otherwise specified)
amiodarone	yes	60	15^c^	100	12
amoxicilin	yes	75	17	250	20
ceftazidime	yes	180	45	500	42
clindamycin	yes	180	45	30	38
clonidine	yes	40	20	15 μg/min	10 μg/ml
dexmedetomidine	yes	120	15	6 μg/min	8 μg/ml
dobutamine	no	2	0	N/A	5
dopamine	no	2	0	N/A	4
epinephrine/ adrenalin	no	2	0	N/A	0.1
esomeprazole	yes	120	100	4	1.6
fentanyl	yes	20	10	25 μg/min	0.05
phenylephrine	no	4	1	15 μg/min	0.1
flucloxacillin	yes	120	30	500	50
furosemide	yes	60	30	20	5.0
gentamycin	yes	120	15	33	1
heparin	yes	15	30	1500 IU/min	400 IU/ml
hydrocortisone	yes	180	90	50	4
insulin	t.b.d.^d^	15	15	0.8 IU/min	1 IU/ml
potassium chloride	yes	60	30	0.3 mmol/min	1 mmol/ml
s-ketamine	yes	10	5	5	5
methylprednisolone	yes	120	90	30	60
magnesium sulfate	yes	60	60	200 mg/min	100 mg/ml
midazolam	yes	15	25	2	2
milrinone	yes	140	30	0.3	0.2
morphine	yes	20	15	4	1
nicardipine	yes	30	30	0.5	1.0
nitroglycerin	no	15	7	0.5	0.5
norepinephrine/ noradrenalin	no	2	0	N/A	0.1
paracetamol	yes	120	60	60	10
propofol	yes	15	4	200	20
sufentanil	yes	30	30	25 μg/min	10 μg/ml
tacrolimus	yes	240	60	7 μg/min	40 μg/ml
tobramycin	yes	120	15	8	6
vancomycin	yes	120	60	10	40

*ICU* intensive care unit, *min* minutes, *BT*_*1/2*_ biological half-life, *IU/ml* International units per milliliter, *N/A* not applicable, since interruption is not allowed

^a^Assessed by clinical experts from our local intensive care unit and hospital pharmacy

^b^Assuming a body weight of > 60 kg

^c^Amiodarone’s maximal interruption time may become longer after multiple days of therapy

^d^To be determined. Insulin is known to adsorb to the tubing wall, future study is required to determine suitability for drug multiplexing

An anonymized database was constructed from 69,730 unique ICU drug administrations retrieved from our adult ICU Patient Data Management System (Metavision, iMDSoft, Tel Aviv, Israel) recorded between March of 2014 and February of 2016 (Additional file 2). Each drug administration was linked to a one-way encrypted patient identifier and documented the type and class of drug, concentration, administration time period, volume and rate of infusion. Since the database contained no personally identifiable data, no ethical approval was required. We included 36 of the most frequently used drugs for which multiplex scheduling parameters were known. Maintenance infusion fluids and total parenteral nutrition were also excluded as this study focused on therapeutic drugs. From the remaining drug administrations, groups of drugs that were administered within the same hour to the same patient were recorded and used as input for the scheduling algorithm.

### Conventional scheduling procedure

**Fig. 2:**
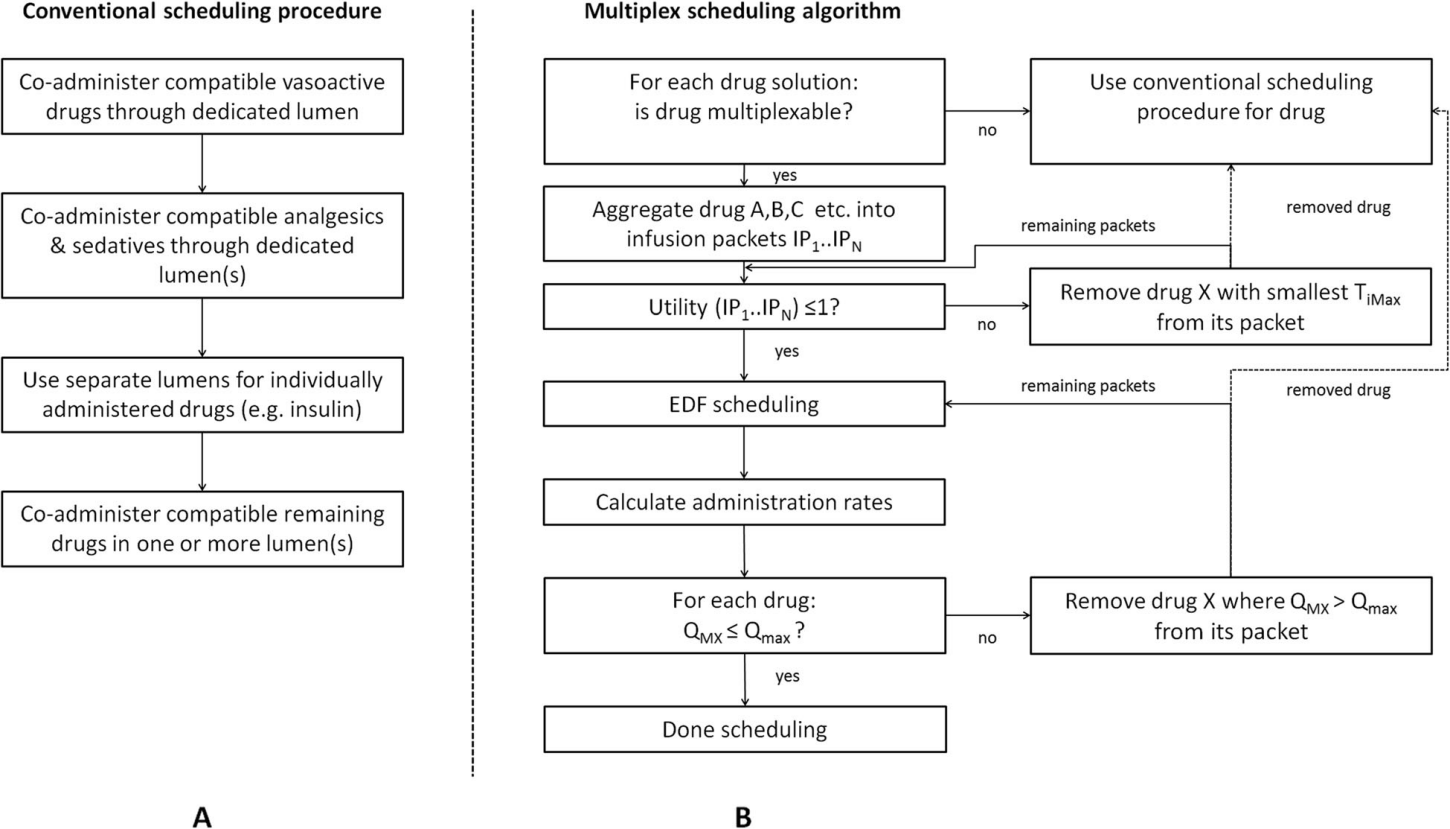
Conventional scheduling procedure and the multiplex scheduling algorithm. Using the conventional scheduling procedure drugs are initially divided lumens based on drug category and subsequently based on compatibility (Panel **a**). The multiplex algorithm (Panel **b**) has to satisfy utility and maximal administration rate (Q_MAX_) related constraints for successful scheduling. When a drug cannot be multiplexed, it will be scheduled following the conventional scheduling procedure.

In order to simulate the conventional method of drug scheduling, local protocols and ICU nurses of our 42-bed tertiary care ICU were consulted. In a semi-structured interview four ICU nurses were asked to describe how they decide which drugs to co-administer. From these interviews combined with our pharmacy protocols we distilled the following procedure (Fig. 2a): First, all vasoactive drugs can be co-administered through a single designated lumen. As most vasoactive drugs are compatible with each other a single lumen is generally sufficient for this purpose. Second, analgesics and sedatives are co-administered through one or more lumens, depending on drug compatibilities. Third, drugs that are preferably not co-administered with other drugs are administered through a dedicated lumen (e.g. insulin). Finally, all other remaining drugs are administered through one or more lumens depending on their compatibilities.

### Multiplex scheduling algorithm

Figure 2b shows a flow chart of the multiplex algorithm. The multiplex scheduling algorithm’s input is a list of drugs to be administered, and a database containing y-site drug compatibilities, whether multiplexing is allowed, and pharmacokinetic and pharmacodynamic (PK/PD) parameters such as biological half-life T_1/2_ and maximal interruption time T_iMax_ (Table 1). The relation between period P_i_ of the infusion packet IP_i_ duration D_i_, and T_iMax_ is defined by the following equation [10]:.

1 $$ {P}_i={D}_i+\frac{1}{2}{T}_{iMax} $$

The multiplex scheduling algorithm initially differentiates between multiplexable and non-multiplexable drugs. Non-multiplexable drugs are scheduled using the conventional scheduling procedure. For the remaining multiplexable drugs the algorithm attempts to combine drugs into packets. An infusion packet IP_i_ is defined as a collection of compatible drugs which are administered simultaneously during multiplex infusion together with the subsequent required volume of a separator fluid (Fig. 1).

The T_iMax_ of an infusion packet IP_i_ is equal to the smallest T_iMax_ of the drugs within that packet, ensuring that for every drug in a packet the T_iMax_ constraint is respected. The D_i_ for a packet will be equal to the sum of the largest administration time of the drugs in packet IP_i_ (D_drugs_i_) and the time for separator fluid administration (D_sep_i_) as shown in Formula 2.

2 $$ {D}_i={D}_{drugs\_i}+{D}_{sep\_i} $$

The value of D_drugs_i_ could be configured in the algorithm, however we did not know its optimal value beforehand. Therefore, we ran the algorithm setting D_drugs_i_ to 1, 2, 5, 10 and 20 min. In our model D_sep_i_ was set to 1 min, which will be sufficient time to flush the tubing in most settings.

The value for P_i_ was calculated using Formula 1. The multiplex algorithm attempts to combine as many drugs as possible within a single packet. However, there is a limit to the number of packets that can be multiplexed without violating T_iMax_ constraints. In order to determine the fraction of use of the IV tube over time a utility value (U) is calculated (Formula 3) [9].

3 $$ U=\sum \limits_{i=1}^n\frac{D_i}{P_i} $$

As an example: For two packets A and B, packet_i_ {D_i_, P_i_} is set to A {2, 3}, and B {1, 4} respectively. The corresponding utility value is $\frac{2}{3}+\frac{1}{4}=\frac{11}{12}\approx 0.92$. A utility value > 1 would indicate that the fraction of use of the IV tube is larger than the capacity of that tube. A utility value ≤1 indicates that the EDF algorithm is able to create an administration schedule that does not violate the T_iMax_ constraints of the packets to be scheduled. It must be noted that in a subsequent stage administration rates will be calculated which are not allowed to exceed the maximally allowable administration rate. Hence a utility value ≤1 is a necessary, but not a final criterion for a multiplex administration schedule. When the utility value is > 1 the algorithm will remove the drug with the smallest value of T_iMax_ from its packet and will schedule that drug as a non-multiplexable drug. For the remaining multiplexable drugs the utility value is recalculated until the utility value is ≤1.

**Fig. 3:**
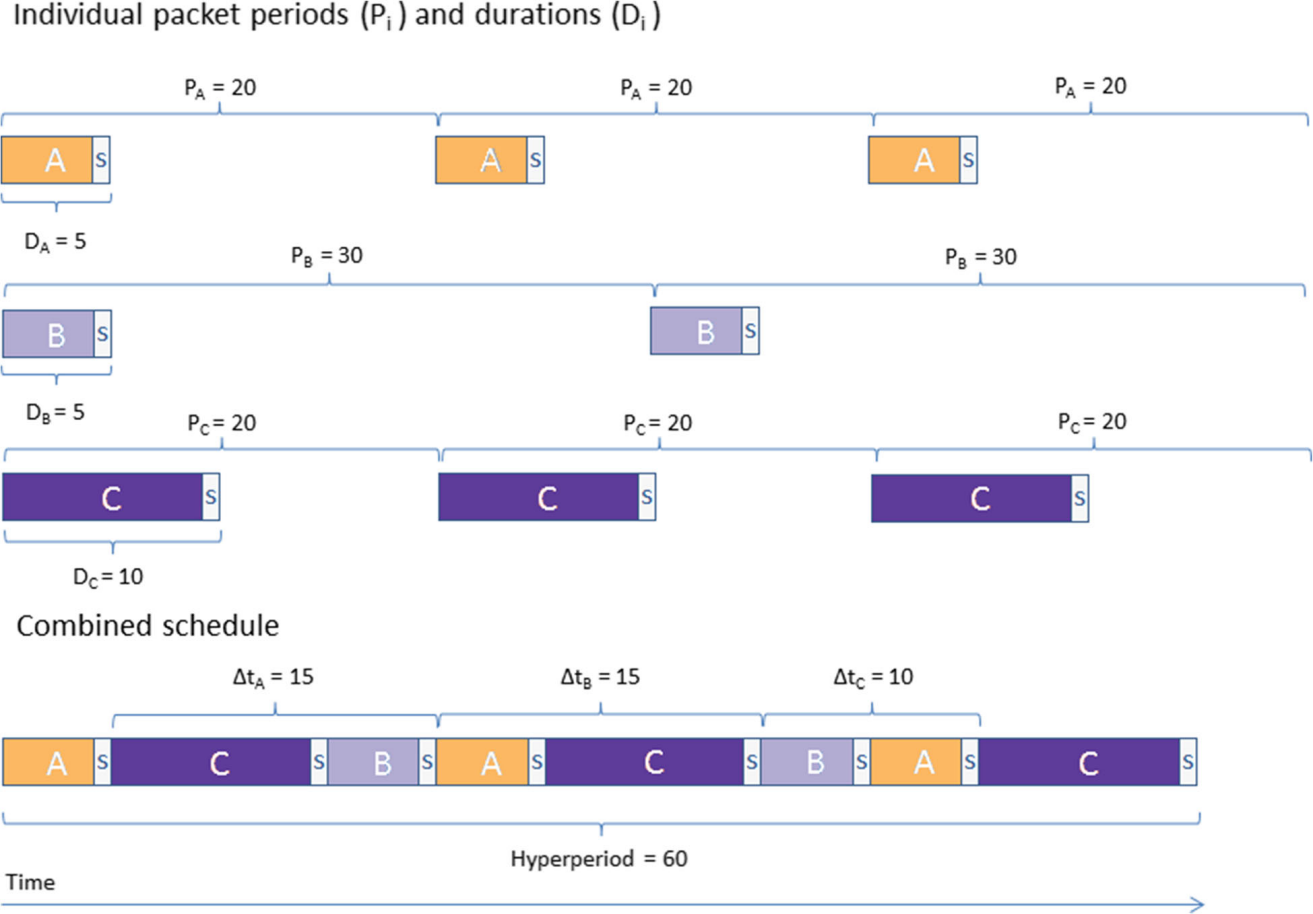
Earliest deadline first (EDF) scheduling. The end of every period P_I_ is a deadline for the administration of the respective packet. A separator packet A separator fluid volume (SFV) is considered as part of each packet during scheduling. The deadline is related to the PK/PD characteristics of the drug or solution so that sufficiently stable sustained biological action of the constituent(s) is maintained under repeated interrupted administration. Here the so called utility, or U-value is U_A_ + U_B_ + U_C_ = 5/20 + 5/30 + 10/20 = 11/12. AS U ≤ 1, Scheduling is feasible. Packet A, that has the nearest deadline, is scheduled first, followed by packets C and B until all Packets are scheduled. The Hyperperiod, or least common mutiple of the periods, is 60 minutes in the example.

The next step in the algorithm is EDF scheduling (Fig. 3) [9]. The constraints for EDF scheduling are the period P_i_ and the packets’ durations D_i_..D_N_. In our application of EDF scheduling the end of each packet’s respective period is considered to be the deadline before which the packet must be scheduled. In the example in Fig. 3 there are three packets A {5, 20}, B {5, 30}, and C {10, 20}. Here the utility U = 5/20 + 5/30 + 10/20 = 11/12. As U ≤ 1, scheduling is feasible. Packet A, that has the nearest deadline, is scheduled first, followed by packets C and B until all packets are scheduled. Each packet will be scheduled only once within a period, and the end of every period is another deadline. The EDF algorithm schedules the packets starting with the packet that has the nearest deadline, and continues until ithe hyperperiod is reached [12].

After scheduling the administration rates were calculated for each packet. The calculation used the conventional administration rate and the available administration time determined by the multiplex algorithm. For example, when drug A was administered at a conventional rate (Q_CONV_) of 5 ml/h over the period of 1 h with an available administration time in the multiplex administration schedule of 20 min, the multiplex administration rate (Q_MX_) then becomes 5 x (60/20) = 15 ml/h. This rate ensures that over a period of 1 h the same volume of A is administered during multiplexing. Q_MX_ is subsequently compared to the maximal administration rate (Table 1). If Q_MX_ is larger than the maximal administration rate, the corresponding drug is removed from its packet and scheduled as a non-multiplexable drug. For the remaining multiplexable drugs the schedule is recalculated.

### Statistical analysis

We defined ΔL as the difference between the number of lumens required by conventional infusion (L_CONV_) and multiplex infusion (L_MX_), i.e. L_CONV_ - L_MX_. In the descriptive statistics the mean and standard deviation (SD) are shown in case of normally distributed data, otherwise the median and interquartile range (IQR) are shown.

Group differences (L_CONV_ vs. L_MX_) were assessed using a pairwise t-test when normally distributed, otherwise the Wilcoxon signed ranks test was used. Finally, regression analysis was performed to determine the relation between the L_CONV_ and ΔL.

## Results

A total of 175,993 drug combinations that were administered to 2715 patients were scheduled using both the conventional procedure and the multiplex algorithm.

**Fig. 4:**
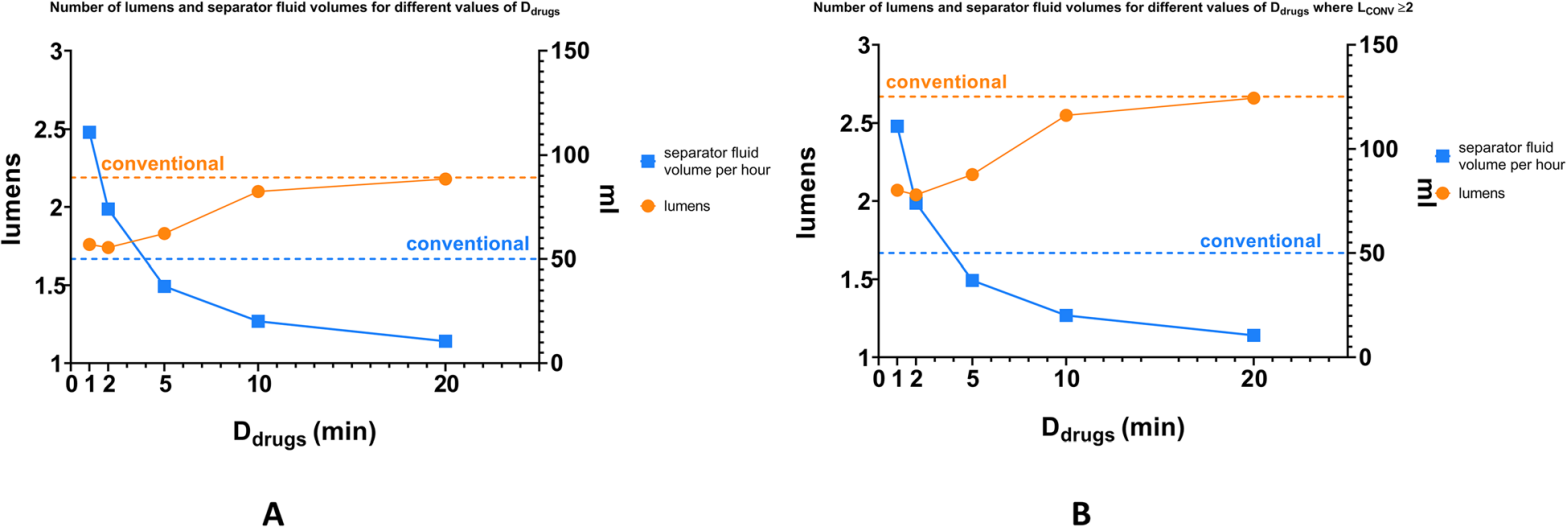
Lumens and separator fluid volumes required by the multiplex algorithm for the different values of D_drugs_. Panel **a** shows lumens and separator fluid volumes for all levels of L_CONV_ assuming a Vygon V-Green IV tube (Vygon, France; 2 m, 2 mL). Panel **b** shows the same data, however schedules where L_CONV_ was equal to 1 were omitted as this number could obviously not be reduced to zero by multiplexing. In both panels the dashed orange line indicates the mean of L_CONV_ and the dashed blue line indicates the mean hourly volume of volumetric saline and glucose infusions

Figure 4a shows a summary of L_MX_ for the different values of D_drugs_ as well as the corresponding separator fluid volume assuming a Vygon V-Green IV tube (Vygon, France; 2 m, 2 mL) which is the default IV tube in our ICU. Figure 4b shows the same data, however schedules where L_CONV_ was equal to 1 were omitted as the number of lumens could not be reduced in these cases.

As setting D_drugs_ to 5 min best suited clinical constraints in the ICU substudy, only the corresponding results are provided in the main text. Complete data for the different values of D_drugs_ are listed in Additional files 3,4, 5, 6, 7 and 8.

**Fig. 5:**
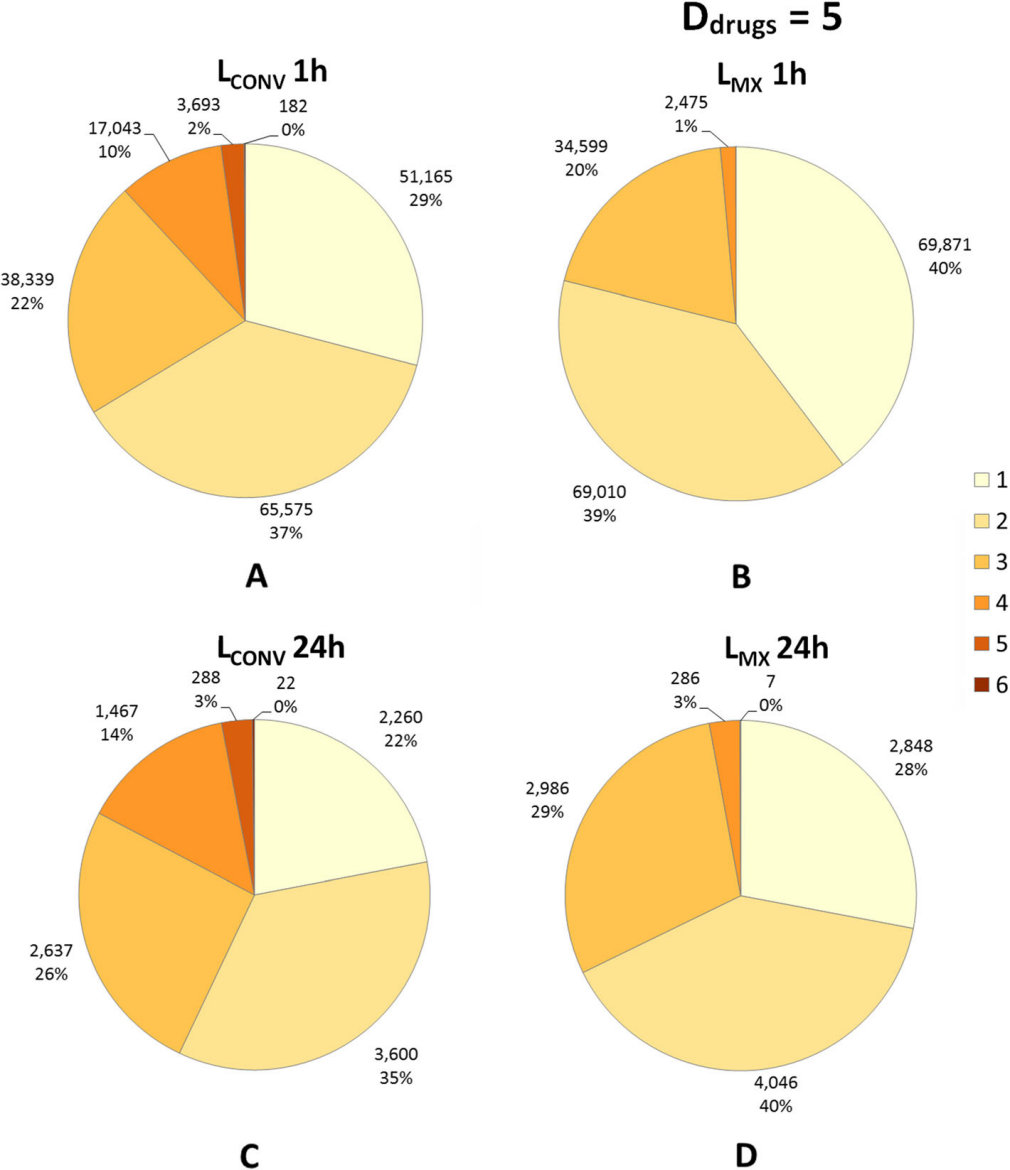
Number of IV lumens required by conventional scheduling (L_CONV_) and multiplex scheduling (L_MX_). Values of L_CONV_ and L_MX_ as determined over 1 h periods (panels **a** and **b**) and the maximal values of L_CONV_ and L_MX_ aggregated over 24 h periods from midnight to midnight (panels **c** and **d**). Note that D_drugs_ = 5 min in panels **b** and **d**

Figure 5 displays the values of L_CONV_ and L_MX_ over 1 h periods (Fig. 5a and b) and maximal values of L_CONV_ and L_MX_ aggregated over 24 h periods (Fig. 5c and d). The median [IQR] of L_CONV_ was significantly higher than that of L_MX_ at both 1 h (2 [1–3] vs. 2 [1–2] respectively, *p* < 0.001) and 24 h periods (2 [2–3] vs. 2 [1–3] respectively, p < 0.001).

The mean ± SD number of IV solutions was 2.8 ± 1.6. In 27% of all drug combinations, or 61% of the 2251 unique combinations, multiplexing could reduce the number of lumens compared to conventional drug administration (i.e. ΔL > =1). Table 2 shows the mean and median L_MX_ for every level of L_CONV_ as well as the frequency distribution of ΔL for every level of L_CONV_. A significant linear regression equation was found (F (1, 175,995) =125,416, p < 0.001), and the predicted value of ΔL was equal to − 0.536 + 0.409 * L_CONV_.

**Table 2:** Relation between levels of L_CONV_, the corresponding values of L_MX_ and the reduction in lumens

Number of conventional lumens (L_**CONV**_)	N	Total number of solutions Mean ± SD	L_MX_^*^ Mean ± SD	L_MX_^*^ Median [IQR]	Reduction in lumens (ΔL) N (%)			P^**^
					ΔL = 1	ΔL = 2	ΔL = 3	
**1**	51,165	1.2 ± 0.4	1.0 ± 0.0	1 [1–1]	0 (0%)	0 (0%)	0 (0%)	not applicable
**2**	65,575	2.5 ± 0.6	1.8 ± 0.4	2 [2–2]	13,831 (21%)	0 (0%)	0 (0%)	< 0.001
**3**	38,339	3.8 ± 0.8	2.5 ± 0.7	3 [2–3]	9298 (24%)	4778 (13%)	0 (0%)	< 0.001
**4**	17,043	5.2 ± 1.0	2.7 ± 0.7	2 [2–3]	7399 (43%)	7326 (43%)	97 (1%)	< 0.001
**5**	3693	6.8 ± 1.0	2.9 ± 0.5	3 [3–3]	166 (5%)	2843 (77%)	642 (17%)	< 0.001
**6**	182	7.5 ± 0.9	3.5 ± 0.5	3 [3–4]	0 (0%)	88 (48%)	94 (52%)	< 0.001

L_MX_: Number of lumens required in a multiplex administration schedule

*SD* Standard deviation, *IQR* Interquartile range

^*^D_drugs_ was set to 5 min

^**^Wilcoxon Signed Ranks test for the difference between the medians of L_CONV_ and L_MX_

## Discussion

In this study we modeled the performance of an algorithm that is designed to reduce the number of IV lumens required for the administration of multiple incompatible drugs. In almost all cases multiplexing required 3 or fewer lumens, which indicates that one triple-lumen central venous catheter would be sufficient for IV drug administration in nearly all ICU patients [13]. This is an important result as this could reduce IV therapy related infections and phlebitis that currently occur in 20–40% of peripheral venous catheters [14–16]. The results also indicate that the more lumens are required in conventional infusion, the larger the reduction in lumens becomes when multiplex infusion is applied.

For many of the drugs in Table 1, the ratio between the possible maximal infusion rates and actual necessary mean infusion rates is very large. Such a large ratio indicates that only a small time fraction is required to safely administer the drug, allowing considerable flexibility for the multiplex algorithm. The original article of Liu and Layland discusses a scheduling algorithm that dynamically assigns priorities to tasks to be performed by a single computer processor [9]. By analogy a single IV tube can be regarded as a sequential processor whose tasks are the administrations of various drug packets which all have their own time constraints. The utility value in the current application must be ≤1, which is a necessary but not sufficient criterion for successful scheduling. In the original EDF algorithm preemptive scheduling was applied, meaning that tasks could be interrupted by a task with a higher priority and resumed at a later moment. This property is very useful in a dynamic real-time environment, however in the current application schedules are calculated before execution instead of in real-time. Therefore, non-preemptive scheduling was applied in this study, meaning that packets were always scheduled for their complete duration without interruption.

In clinical practice it will be a common scenario that fluids are added or removed from a multiplex administration schedule. In such cases the multiplex algorithm will recalculate a new administration schedule using the updated fluid selection. The workflow for nurses using multiplex infusion will be somewhat different from that of conventional infusion when it comes to arranging the IV tubing. For example, when adding a drug to an existing multiplex administration schedule the nurse will deliberately connect a drug to the tubing of one or more incompatible fluids. We are currently designing and testing a user-interface that safely and intuitively guides the nurse through the necessary steps. The changing of administration rates during multiplexing will be largely similar to changing a conventional (continuous) rate as long as the rate does not exceed the maximal allowable rate, which are quite high for many drugs (Table 1). Equivalent to conventional IV therapy, nurses must always be vigilant to risks of air in line or occlusions when multiplexing [17].

The maximal interruption times in Table 1 were determined in a parallel research project. Solutions were included that (according to published literature) were commonly administered continuously and intermittently. For these solutions the target blood concentrations were determined by modelling the steady state concentrations during continuous IV administration over a 24 h period using population pharmacokinetic models available in literature and the MWPharm software package [18]. During simulated multiplexing, the blood level concentration was allowed to deviate by maximally ±10% from the target concentration - which is quite a conservative limit - as simulated by MWPharm. This limit was determined analogous to the ±10% deviation limit in the Dutch law for drug preparations [19]. This in turn allowed the determination of the maximal interruption time. Finally, an expert panel consisting of intensivists and pharmacists reviewed the maximal interruption times, a process where also PD knowledge on the clinical duration of action of drugs was taken into account. In the case of disagreement between the experts the most conservative estimate of T_iMax_ was used. For various reasons other healthcare facilities may prefer using different scheduling parameters. In such a case the multiplex scheduling algorithm is versatile enough to use these different parameters to create a feasible administration schedule.

The multiplex scheduling algorithm was tested using different values for the duration of drug administrations within a packet (D_drugs_). There was a trade-off between the value of D_drugs_ and the required volume of separator fluid (Fig. 4). At low values of D_drugs_, drugs with a low T_iMax_ were more likely to be scheduled, however a large volume of separator fluid was required as there are many alternations between the packets. At a high value of D_drugs_ less separator fluid was required, however some drugs with low a T_iMax_ could not be scheduled. In a clinical situation the start-up delay of infusion pumps must be taken into account as it may lead to an administered volume that deviates from the targeted volume at too low values of D_drugs_ (e.g. < 2 min) [19, 20–22]. Overall lower D_drugs_ values corresponded to a lower L_MX_, and higher administration rates relative to conventional drug administration (Additional file 9). At very high values of D_drugs_ (e.g. ≥ 10 min) the advantage of multiplexing compared to conventional drug administration was negligible (Additional files 6 and 7).

With respect to the solution that serves as separator fluid, the duration D_sep_ will depend on the required separator fluid volume (SFV) and its maximal allowable administration rate. The SFV in turn depends on the shared infusion volume (the volume of the tubing through which all multiplexed fluids pass; SIV). A previous study indicated that, for a standard IV tube as is used in our ICU (Vygon, France; 2 m, 2 mL), a SFV of 3.7 mL is required to prevent mixing of two subsequent packets [23]. As a rule of thumb, twice the SIV must be flushed to sufficiently separate of two packets. Considering that the administration rate of modern syringe pumps can often be set at up to 500 ml/h we believe that setting D_sep_i_ to 1 min is reasonable.

With a D_drugs_ of 5 min and using a standard (2 m, 2 mL) IV tube, approximately 1.1 L of separator fluid would be required per patient per day. As an average patient in our ICU receives 1.2 L in volumetric saline and glucose infusions per day, these could also be used as separator fluid. Reducing the SIV to 1 mL, will require approximately 0.55 L of separator fluid per day. This may be especially convenient in patients who are treated using a restricted fluid regimen, such as patients with acute respiratory distress syndrome [24, 25]. Other drug solutions may also serve as separator fluid when they are compatible with the drugs in surrounding packets. Drug dose and administration rate limits will be important constrains in such a case and it will require further study to assess the feasibility of this concept.

It must be noted that in this study central venous pressure (CVP) measurements were not taken into account, which may require a dedicated central lumen in some hospital settings. Likewise it may be desirable to have a separate lumen available for drawing blood samples [26]. During multiplexing it may be a useful feature to schedule empty packets where no drug administration takes place, allowing for periodic CVP measurements or blood draws without the need of an additional lumen. Boluses and intermittent infusions were also not taken into account. In the case where there is no lumen available, the multiplex administration schedule should be flexible enough to quickly clear (flush) the IV tube to allow a higher priority infusion. Subsequently the system should be able to resume with a (modified) multiplex schedule. The multiplex algorithm did not take a preferred vascular access site into account. Although multiplexing is most easily performed for central venous access, this is not required.

There are many degrees of freedom in the multiplex algorithm. D_drugs_, D_sep_, and the scheduling parameters in Table 1 all affect the value of L_MX_. Therefore, L_MX_ may differ in situations where clinicians have other preferences or constraints. The drugs used in this study were among the most commonly used drugs in our ICU, which may be different from other ICUs or other departments where multi-infusion takes place. Fluids that are not yet present in the multiplex database will be considered incompatible with all other fluids. Likewise, drugs with unknown scheduling parameters (e.g. undetermined T_iMax_) will not be multiplexed. Further studies would be required to add the currently unknown scheduling parameters of those drug solutions to our database. Nevertheless, the use of our top 36 of drugs covered almost 97% of all IV drug administrations in our ICU.

## Conclusion

The multiplex algorithm tackles an important issue in ICUs when several incompatible intravenous drugs have to be administered through a limited number of lumens. The multiplex algorithm requires fewer IV lumens compared to the conventional procedure.

## Supplementary information

**Additional file 1.** Compatibility chart.

**Additional file 2.** Flow of study data.

**Additional file 3.** Relation between levels of L_CONV_, the corresponding values of L_MX_ and the reduction in lumens where D_drugs_ = 1 min.

**Additional file 4.** Relation between levels of L_CONV_, the corresponding values of L_MX_ and the reduction in lumens where D_drugs_ = 2 min.

**Additional file 5.** Relation between levels of L_CONV_, the corresponding values of L_MX_ and the reduction in lumens where D_drugs_ = 5 min.

**Additional file 6.** Relation between levels of L_CONV_, the corresponding values of L_MX_ and the reduction in lumens where D_drugs_ = 10 min.

**Additional file 7.** Relation between levels of L_CONV_, the corresponding values of L_MX_ and the reduction in lumens where D_drugs_ = 20 min.

**Additional file 8.** Number of IV lumens required by multiplex scheduling (L_MX_; panels A-E and G-K) and by conventional scheduling (L_conv_; panels F and L). Values of L_conv_ and L_mx_ were determined over 1 h periods (panels A-F) and the maximal values of L_conv_ and L_mx_ aggregated over 24 h periods from midnight to midnight (panels G and L). Note that for D_drugs_ = 1, 2, and 5 a maximum of 4 lm is required, whereas for D_drugs_ = 10 and 20 a maximum number of 6 lm is required.

**Additional file 9.** Relation between the multiplex administration rate (Q_MX_) and the conventional rate (Q_CONV_) for different values of D_drugs_.

## Data Availability

The datasets used and/or analyzed during the current study are available from the corresponding author on reasonable request.

## Abbreviations

dL or ΔL: Difference in IV lumens required by multiplex and conventional procedure.
CI: Confidence interval
CVC: Central venous catheter
CVP: Central venous pressure
D_drugs_i_: Duration of the drug administration within Packet i
D_i_: Duration of Packet i, including the administration of drugs and separator fluid
D_sep_i_: Duration of the administration of a separator fluid that is part of packet i
EDF: Earliest deadline first
ICU: Intensive care unit
BT_1/2_: Half-life of the biological effect
T_iMax_: Maximal interruption time
IV: Intravenous
IP_i_: Infusion packet i
L_CONV_: Number of lumens required in a conventional administration schedule
L_MX_: Number of lumens required in a multiplex administration schedule
MX-IS: Multiplex infusion system
P_i_: Period of infusion packet i
PD: Pharmacodynamic(s)
PK: Pharmacokinetic(s)
PVC: Peripheral venous catheter
Q_max_: The maximally allowable (bolus) administration rate in mg/min
SD: Standard deviation
SIV: Shared infusion volume
SFV: Separator fluid volume
T_iMax_: Maximal interruption time
U_i_: Utility of packet i
