# Supplementary material for: Towards more efficient use of intravenous lumens in multi-infusion settings: development and evaluation of a multiplex infusion scheduling algorithm
Source: BMC Med Inform Decis Mak. 2020 Sep 2;20:206. doi: 10.1186/s12911-020-01231-w (PMC7466776; doi:10.1186/s12911-020-01231-w)
Supplement: Supplementary file 1 — Additional file 1. Compatibility chart. [file 12911_2020_1231_MOESM1_ESM.pdf]

Additional file 1. Compatibility chart.

	AMIODARONE	ARGIPRESSIN	BENZYLPENICILLIN-SODIUM	BUMETANIDE	CEFTAZIDIME	CLINDAMYCINE	CLONIDINE	DEXMEDETOMIDINE	DOBUTAMINE	DOPAMINE	ESKETAMINE	FLUCLOXACILLIN	FUROSEMIDE	HEPARIN	HYDROCORTISONE	INSULIN	ISOPRENALINE	POTASSIUM CHLORIDE	LABETALOL	MAGNESIUM SULFATE	MIDAZOLAM	MILRINONE	MORPHINE	SODIUM PHOSPHATE	NICARDIPINE	NITROGLYCERIN	NITROPRUSSIDE	NOREPINEPHRINE	OCTREOTIDE	PANTOPRAZOLE	PROPOFOL	ROCURONIUM	TACROLIMUS	VANCOMYCIN	
AMIODARONE		C	U	C	I	C	U	C	C	C	C	U	U	I	I	U	C	C	C	U	C	C	C	I	C	C	U	U	C	U	U	C	C	C	AMIODARONE
ARGIPRESSIN	C		U	C	C	C	U	C	C	C	U	U	U	C	C	U	C	C	C	C	C	C	C	U	C	C	C	C	C	C	U	C	C	C	ARGIPRESSIN
BENZYLPENICILLIN-SODIUM	U	U		C	C	C	U	U	I	C	U	U	C	C	C	C	C	C	I	C	C	U	C	U	U	C	C	C	U	C	U	U	U	U	BENZYLPENICILLIN-SODIUM
BUMETANIDE	C	C	C		C	C	U	C	C	C	U	C	C	C	C	C	C	C	C	C	U	U	C	U	C	C	C	C	C	C	C	C	C	C	BUMETANIDE
CEFTAZIDIME	I	C	C	C		C	U	C	U	C	C	C	C	C	C	C	C	C	C	C	I	C	C	U	U	C	I	C	C	U	U	C	C	U	CEFTAZIDIME
CLINDAMYCINE	C	C	C	C	C		U	C	C	C	C	U	C	C	C	C	C	C	U	C	U	C	C	U	U	C	C	C	C	U	C	C	C	C	CLINDAMYCINE
CLONIDINE	U	U	U	U	U	U		U	C	C	C	U	U	C	U	U	C	C	C	C	U	U	C	U	U	C	U	C	U	U	U	U	U	U	CLONIDINE
DEXMEDETOMIDINE	C	C	U	C	C	C	U		C	C	U	U	C	C	C	C	C	C	C	C	C	C	C	C	C	C	C	C	C	I	C	C	C	C	DEXMEDETOMIDINE
DOBUTAMINE	C	C	I	C	U	C	C	C		C	C	U	I	U	I	U	C	C	C	C	I	C	C	U	C	C	C	C	C	I	U	C	C	C	DOBUTAMINE
DOPAMINE	C	C	C	C	C	C	C	C	C		C	U	U	C	C	I	C	C	C	C	C	C	C	U	C	C	C	C	C	U	U	C	C	C	DOPAMINE
ESKETAMINE	C	U	U	U	C	C	C	U	C	C		U	I	I	C	I	U	C	U	C	C	C	C	U	U	U	U	U	U	U	C	U	U	C	ESKETAMINE
FLUCLOXACILLIN	U	U	U	C	C	U	U	U	U	U	U		U	C	C	U	C	C	U	U	I	U	U	U	U	U	U	U	U	U	U	U	U	I	FLUCLOXACILLIN
FUROSEMIDE	U	U	C	C	C	C	U	C	I	U	I	U		C	C	U	U	C	U	U	C	I	U	U	I	C	C	U	C	C	C	I	U	I	FUROSEMIDE
HEPARIN	I	C	C	C	C	C	C	C	U	C	I	C	C		C	U	C	C	I	C	C	C	C	U	U	C	C	C	C	U	C	C	C	U	HEPARIN
HYDROCORTISONE	I	C	C	C	C	C	U	C	I	C	C	C	C	C		C	C	C	I	U	I	C	C	U	I	C	C	C	C	I	C	I	C	U	HYDROCORTISONE
INSULIN	U	U	C	C	C	C	U	C	U	I	I	U	U	U	C		I	C	I	C	U	C	C	U	U	C	C	U	C	U	C	I	C	C	INSULIN
ISOPRENALINE	C	C	C	C	C	C	C	C	C	C	U	C	U	C	C	I		C	C	C	C	C	C	U	C	C	C	C	C	U	C	C	C	C	ISOPRENALINE
POTASSIUM CHLORIDE	C	C	C	C	C	C	C	C	C	C	C	C	C	C	C	C	C		C	C	C	C	C	U	C	C	C	C	C	C	C	C	C	C	POTASSIUM CHLORIDE
LABETALOL	C	C	I	C	C	U	C	C	C	C	U	U	U	I	I	I	C	C		C	C	C	C	U	C	C	C	C	C	I	C	C	C	C	LABETALOL
MAGNESIUM SULFATE	U	C	C	C	C	C	C	C	C	C	C	U	U	C	U	C	C	C	C		C	C	C	U	C	C	C	C	C	U	C	C	C	C	MAGNESIUM SULFATE
MIDAZOLAM	C	C	C	U	I	U	U	C	I	C	C	I	C	C	I	U	C	C	C	C		C	C	U	C	C	C	C	C	U	C	C	C	C	MIDAZOLAM
MILRINONE	C	C	U	U	C	C	U	C	C	C	C	U	I	C	C	C	C	C	C	C	C		C	C	C	C	C	C	C	I	C	C	C	C	MILRINONE
MORPHINE	C	C	C	C	C	C	C	C	C	C	C	U	U	C	C	C	C	C	C	C	C	C		U	C	C	C	C	C	U	U	C	C	C	MORPHINE
SODIUM PHOSPHATE	I	U	U	U	U	U	U	C	U	U	U	U	U	U	U	U	U	U	U	U	U	C	U		C	U	U	U	C	I	U	C	C	U	SODIUM PHOSPHATE
NICARDIPINE	C	C	U	C	U	U	U	C	C	C	U	U	I	U	I	U	C	C	C	C	C	C	C	C		C	C	C	C	I	U	C	C	C	NICARDIPINE
NITROGLYCERIN	C	C	C	C	C	C	C	C	C	C	U	U	C	C	C	C	C	C	C	C	C	C	C	U	C		C	C	C	U	U	C	C	C	NITROGLYCERIN
NITROPRUSSIDE	U	C	C	C	I	C	U	C	C	C	U	U	C	C	C	C	C	C	C	C	C	C	C	U	C	C		C	C	U	C	C	C	C	NITROPRUSSIDE
NOREPINEPHRINE	U	C	C	C	C	C	C	C	C	C	U	U	U	C	C	U	C	C	C	C	C	C	C	U	C	C	C		C	U	C	U	C	C	NOREPINEPHRINE
OCTREOTIDE	C	C	U	C	C	C	U	C	C	C	U	U	C	C	C	C	C	C	C	C	C	C	C	C	C	C	C	C		U	U	C	C	C	OCTREOTIDE
PANTOPRAZOLE	U	C	C	C	U	U	U	I	I	U	U	U	C	U	I	U	U	C	I	U	U	I	U	I	I	U	U	U	U		U	I	U	C	PANTOPRAZOLE
PROPOFOL	U	U	U	C	U	C	U	C	U	U	C	U	C	C	C	C	C	C	C	C	C	C	U	U	U	U	C	C	U	U		U	U	U	PROPOFOL
ROCURONIUM	C	C	U	C	C	C	U	C	C	C	U	U	I	C	I	I	C	C	C	C	C	C	C	C	C	C	C	U	C	I	U		C	U	ROCURONIUM
TACROLIMUS	C	C	U	C	C	C	U	C	C	C	U	U	U	C	C	C	C	C	C	C	C	C	C	C	C	C	C	C	C	U	U	C		C	TACROLIMUS
VANCOMYCIN	C	C	U	C	U	C	U	C	C	C	C	I	I	U	U	C	C	C	C	C	C	C	C	U	C	C	C	C	C	C	U	U	C		VANCOMYCIN
	AMIODARONE	ARGIPRESSIN	BENZYLPENICILLIN-SODIUM	BUMETANIDE	CEFTAZIDIME	CLINDAMYCINE	CLONIDINE	DEXMEDETOMIDINE	DOBUTAMINE	DOPAMINE	ESKETAMINE	FLUCLOXACILLIN	FUROSEMIDE	HEPARIN	HYDROCORTISONE	INSULIN	ISOPRENALINE	POTASSIUM CHLORIDE	LABETALOL	MAGNESIUM SULFATE	MIDAZOLAM	MILRINONE	MORPHINE	SODIUM PHOSPHATE	NICARDIPINE	NITROGLYCERIN	NITROPRUSSIDE	NOREPINEPHRINE	OCTREOTIDE	PANTOPRAZOLE	PROPOFOL	ROCURONIUM	TACROLIMUS	VANCOMYCIN	

code	meaning	instructions
C	Compatible	These drugs can be safely co-administered through a single lumen.
I	Incompatible	These drugs can NOT be co-administered through a single lumen.
U	Unknown	The compatibility of these drugs is unknown. Call the hospital pharmacy for advice.
