# Supplementary figures and images for: Towards more efficient use of intravenous lumens in multi-infusion settings: development and evaluation of a multiplex infusion scheduling algorithm
Source: BMC Med Inform Decis Mak. 2020 Sep 2;20:206. doi: 10.1186/s12911-020-01231-w (PMC7466776; doi:10.1186/s12911-020-01231-w)

**Additional file 2. Flow of study data.**

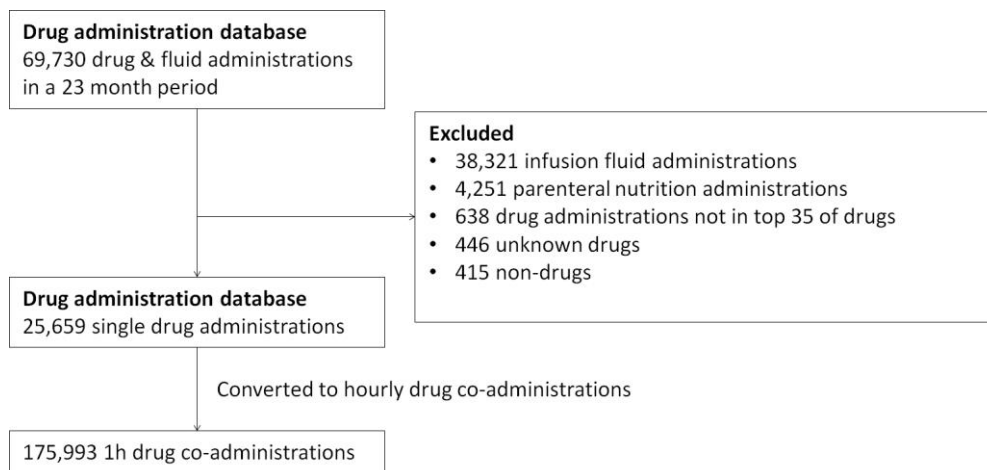

Supplement: Supplementary file 2 — Additional file 2. Flow of study data. [file 12911_2020_1231_MOESM2_ESM.pdf]
