# Supplementary material for: Towards more efficient use of intravenous lumens in multi-infusion settings: development and evaluation of a multiplex infusion scheduling algorithm
Source: BMC Med Inform Decis Mak. 2020 Sep 2;20:206. doi: 10.1186/s12911-020-01231-w (PMC7466776; doi:10.1186/s12911-020-01231-w)
Supplement: Supplementary file 7 — Additional file 7. Relation between levels of LCONV, the corresponding values of LMX and the reduction in lumens where Ddrugs = 20 min. [file 12911_2020_1231_MOESM7_ESM.pdf]

Additional file 7. Relation between levels of L_{CONV}, the corresponding values of L_{MX} and the reduction in lumens where D_{drugs} = 20 minutes

Number of conventional lumens (L_{CONV})	N	Total number of solutions Mean ±SD	L_{MX} Mean ±SD	L_{MX} Median [IQR]	Reduction in lumens (ΔL) N (%)				P*
					ΔL = 1	ΔL = 2	ΔL = 3	ΔL = 4	
**1**	51,165	1.2 ± 0.4	1.0 ± 0.0	1 [1 - 1]	0 (0%)	0 (0%)	0 (0%)	0 (0%)	not applicable
**2**	65,575	2.5 ± 0.6	2.0 ± 0.0	2 [2 - 2]	86 (0%)	0 (0%)	0 (0%)	0 (0%)	<0.001
**3**	38,339	3.8 ± 0.8	3.0 ± 0.1	3 [3 - 3]	362 (1%)	0 (0%)	0 (0%)	0 (0%)	<0.001
**4**	17,043	5.2 ± 1.0	4.0 ± 0.2	4 [4 - 4]	786 (95%)	12 (5%)	0 (0%)	0 (0%)	<0.001
**5**	3,693	6.8 ± 1.0	4.9 ± 0.5	5 [5 - 5]	307 (8%)	128 (4%)	0 (0%)	0 (0%)	<0.001
**6**	182	7.5 ± 0.9	6.0 ± 0.2	6 [6 - 6]	0 (0%)	0 (0%)	181 (99%)	1 (1%)	0.317

L_{MX}: Number of lumens required in a multiplex administration schedule

SD: Standard deviation

IQR: Interquartile range

***Wilcoxon Signed Ranks test for the difference between the medians of L_{CONV} and L_{MX}.**
