# Supplementary material for: Towards more efficient use of intravenous lumens in multi-infusion settings: development and evaluation of a multiplex infusion scheduling algorithm
Source: BMC Med Inform Decis Mak. 2020 Sep 2;20:206. doi: 10.1186/s12911-020-01231-w (PMC7466776; doi:10.1186/s12911-020-01231-w)

Additional file 8. Number of IV lumens required by multiplex scheduling (L_{MX}; panels A-E and G-K) and by conventional scheduling (L_{conv}; panels F and L). Values of L_{conv} and L_{mx} were determined over 1 hour periods (panels A-F) and the maximal values of L_{conv} and L_{mx} aggregated over 24 hour periods from midnight to midnight (panels G and L). Note that for D_{drugs}=1, 2, and 5 a maximum of 4 lumens is required, whereas for D_{drugs}=10 and 20 a maximum number of 6 lumens is required.

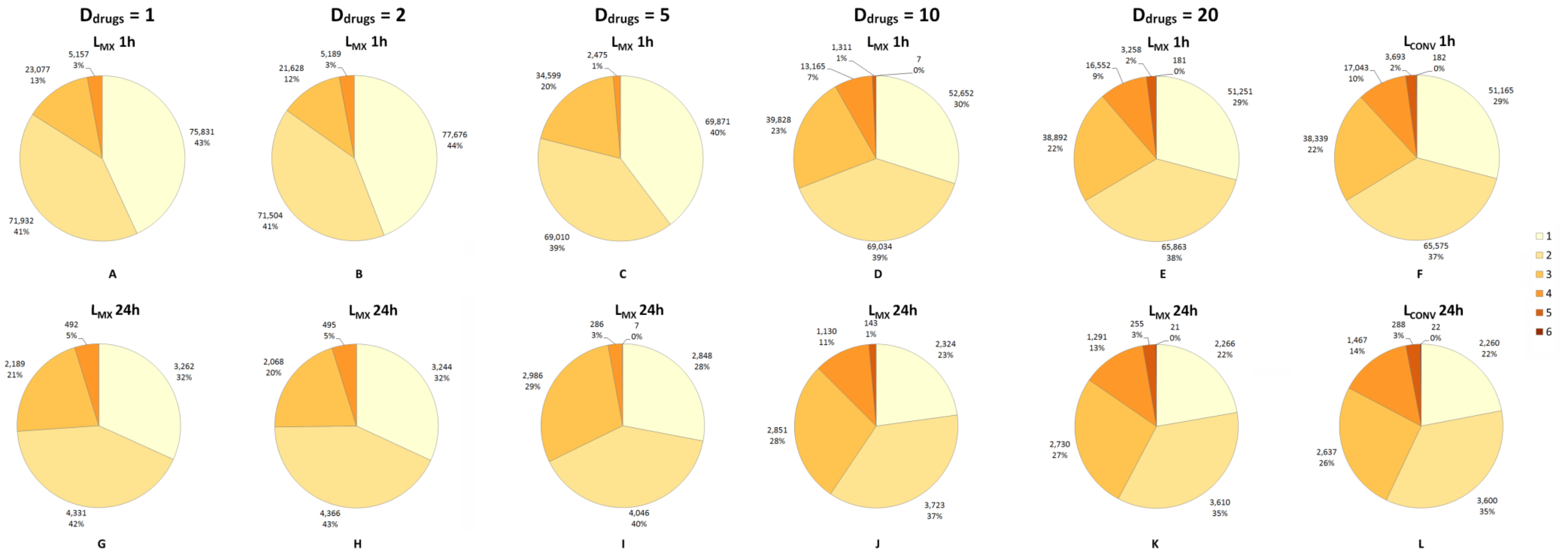

Supplement: Supplementary file 8 — Additional file 8. Number of IV lumens required by multiplex scheduling (LMX; panels A-E and G-K) and by conventional scheduling (Lconv; panels F and L). Values of Lconv and Lmx were determined over 1 h periods (panels A-F) and the maximal values of Lconv and Lmx aggregated over 24 h periods from midnight to midnight (panels G and L). Note that for Ddrugs = 1, 2, and 5 a maximum of 4 lm is required, whereas for Ddrugs = 10 and 20 a maximum number of 6 lm is required. [file 12911_2020_1231_MOESM8_ESM.pdf]
