# Supplementary material for: Towards more efficient use of intravenous lumens in multi-infusion settings: development and evaluation of a multiplex infusion scheduling algorithm
Source: BMC Med Inform Decis Mak. 2020 Sep 2;20:206. doi: 10.1186/s12911-020-01231-w (PMC7466776; doi:10.1186/s12911-020-01231-w)
Supplement: Supplementary file 9 — Additional file 9. Relation between the multiplex administration rate (QMX) and the conventional rate (QCONV) for different values of Ddrugs. [file 12911_2020_1231_MOESM9_ESM.pdf]

Additional file 9. Relation between the multiplex administration rate (Q_{MX}) and the conventional rate (Q_{CONV}) for different values of D_{drugs}.

**Drug name**	**D_{drugs}**	**N**	**Q_{MX} / Q_{CONV} [a] mean ±SD %**
amiodarone	1	4,352	424.5 ±79.5
	2	4,393	312.2 ±38
	5	4,097	240 ±0.0
	10	311	220 ±0.0
amoxicilin	1	416	686.1 ±180.1
	2	416	404.6 ±71.9
	5	384	239.8 ±0.3
	10	5	219.6 ±0.0
clindamycin	10	13	220 ±0.0
clonidine	1	7,345	574.4 ±195.1
	2	7,365	402.7 ±142.8
	5	6,506	240.1 ±2.0
	10	3,382	219.9 ±3.8
dexmedetomidine	1	544	400.4 ±8.6
	2	1,270	300.1 ±2.8
	5	1,071	240 ±0.0
	10	5	220 ±0.0
esomeprazole	1	937	423.8 ±81.5
	2	937	326.1 ±79.7
	5	842	241.1 ±16.5
	10	422	221 ±15.1
	20	761	210 ±0.0
fentanyl	1	103	419.4 ±86.4
	2	103	300 ±0.0
	5	134	240 ±0.0
	10	7	220 ±0.0
flucloxacillin	1	7,026	518.9 ±183.2

	2	7,026	342.7 ±103.8
	5	6,670	240.9 ±14.2
	10	5,780	219.4 ±7.8
	20	64	210 ±0.0
furosemide	1	8,162	452.9 ±141.8
	2	8,230	337.1 ±94.5
	5	8,087	241.5 ±18.2
	10	7,174	220 ±2.0
	20	24	210 ±0.0
heparin	1	2,927	438.4 ±99.1
	2	3,006	382.3 ±120.8
	5	2,928	241.6 ±19.3
	10	2,727	219.8 ±4.7
	20	23	210 ±0.0
insulin	1	43,601	433.1 ±106.2
	2	43,658	303.7 ±21.9
	5	40,702	240 ±0.2
	10	58	219.9 ±0.3
magnesium sulfate	1	14,412	430.1 ±98.9
	2	17,107	307.1 ±36.2
	5	11,536	240.6 ±11.4
	10	9,164	219.9 ±5.2
	20	2,975	210 ±0.4
midazolam	1	7,793	452.9 ±125.9
	2	9,887	307.7 ±31.9
	5	11,682	240 ±0.1
	10	12,018	219.9 ±2.2
	20	109	210 ±0.1
milrinone	1	3,688	421.9 ±81
	2	4,061	307.4 ±33.3

	5	3,234	240.1 ±0.4
	10	4,135	219.6 ±6.7
	20	2	210 ±0.0
morphine	1	15,696	446.7 ±121.9
	2	20,830	304 ±21.6
	5	20,497	240 ±0.1
	10	542	220 ±0.1
nicardipine	1	6,962	443.7 ±121.7
	2	7,580	313.2 ±57.2
	5	6,934	240.9 ±14.4
	10	5,775	219.4 ±7.4
	20	20	210 ±0.0
potassium chloride	1	25,619	434.5 ±111.9
	2	33,003	309.9 ±50.7
	5	26,489	240.1 ±4.1
	10	30,113	219.7 ±5.8
	20	143	210.1 ±0.1
propofol	1	24,641	401.2 ±16.1
	2	27,781	299.6 ±6.4
s-ketamine	1	939	399.1 ±9.2
	2	736	300 ±0.0
	5	6	240 ±0.0
tacrolimus	1	1,481	464.6 ±135.1
	2	1,481	318.6 ±56.7
	5	1,547	239.9 ±12.7
	10	1,019	226.1 ±7.2
	20	1,405	202.7 ±6.7
vancomycin	1	3,046	409.4 ±43.6
	2	4,215	306.7 ±32.2
	5	4,662	240.2 ±7.1

	10	2,977	220 ±0.0
	20	1,428	210 ±0.0
**all**	**1**	**179,671**	**440.1 ±117.7**
	**2**	**203,066**	**313.2 ±56.4**
	**5**	**158,008**	**240.3 ±7.7**
	**10**	**85,627**	**219.8 ±5.4**
	**20**	**6,954**	**208.5 ±4.2**

[a]Q_{MX}: administration rate during a multiplex drug administration; Q_{CONV}: administration rate during conventional drug administration; SD: standard deviation
